# Unravelling the influence of mixed layer depth on chlorophyll-a dynamics in the Red Sea

**DOI:** 10.1371/journal.pone.0318214

**Published:** 2025-03-05

**Authors:** Marianthi Pateraki, Dionysios E. Raitsos, George Krokos, Iason Theodorou, Ibrahim Hoteit

**Affiliations:** 1 Earth Science and Engineering Department, King Abdullah University of Science and Technology, Thuwal, Saudi Arabia; 2 Department of Biology, National and Kapodistrian University of Athens, Athens, Greece; 3 Institute of Oceanography, Hellenic Centre for Marine Research, Anavyssos, Greece; Universidade de Aveiro, PORTUGAL

## Abstract

Primary production in highly stratified and oligotrophic tropical seas relies primarily on nutrient injections from a deepened mixed layer. The Red Sea, one of the warmest marine ecosystems on earth, has very few external nutrient sources. The role of mixed layer depth (MLD) on phytoplankton dynamics has predominantly been investigated in the northern part of the basin, yet a comprehensive investigation covering the entire basin is currently lacking. By integrating numerical MLD simulations and ocean colour remote sensing observations, both regionally-tuned to the Red Sea environment, the influence of vertical mixing, proxied by the MLD, on chlorophyll-a concentration (CHL) is investigated at seasonal and interannual scales. Results show that the central basin exhibits weak relationships, possibly linked to the intense mesoscale activity and the resulting horizontal advective fluxes. Remarkably, in the southern basin, even minor MLD variations (3%) seem to have a significant response in CHL (~10%). Until now, phytoplankton biomass in the south was linked to the horizontal intrusion of nutrient-rich waters from the Indian Ocean, while our results also stress the importance of vertical mixing in the redistribution of these fertile deeper layer waters to the surface lit zone. Here, we report the diverse role of deepened mixed layers in shaping CHL concentrations across various provinces in the Red Sea.

## Introduction

Phytoplankton biomass, as indexed by chlorophyll-a concentration (CHL), [1,2] is considered to be one of the most important marine ecological indicators. It lies at the base of the marine food web and plays a key role in the ocean biological carbon pump (OBCP), facilitating the transfer of organic carbon from the surface to deeper ocean and up the food chain, ultimately altering the Earth’s carbon cycle [3,4]. Understanding the processes controlling nutrient and light distribution, the key components in primary production [5], is therefore important, particularly as oceanic warming was suggested to decrease net primary productivity in the tropics [6]. Since the deeper layers are richer in nutrients, vertical mixing is one of the main mechanisms entraining nutrients to the illuminated surface layer through upwelling or entrainment from the base of the well-mixed surface layer [7].

In tropical seas, where increased thermal stratification limits the accessibility to nutrient-rich deeper layers, the surface layer often exhibits low phytoplankton biomass levels [8]. In these regions, where light is abundant, primary production is primarily limited by nutrient availability [9] and thus, phytoplankton growth highly depends on the upward flux of nutrients from a deepened mixed layer [10], or other nutrient sources like riverine discharge, rainfall and atmospheric dust [11].

However, in the tropical Red Sea external nutrient input is scarce due to the negligible freshwater input and precipitation [12]. Despite the attempt to quantify dust deposition from the adjacent deserts of the Arabian Peninsula [13], its impact on the biogeochemical cycles and marine productivity in the Red Sea remains understudied and uncertain [14]. Nutrient export from the productive coral reef banks has been proposed to play a role in enhancing surface CHL concentrations [15–17], but the degree of this export is highly uncertain [15,18]. Therefore, the only remaining significant nutrient sources in the Red Sea are the deep basin and the water exchange with the Indian Ocean through the Gulf of Aden, via the Bab Al Mandeb strait at the southern end of the basin [19]. As a consequence, the oligotrophic Red Sea exhibits higher phytoplankton biomass at its southern end, attributed to the intrusion of nutrient-rich Gulf of Aden waters [20,21].

In spite of being one of the warmest, most saline and stratified bodies of water [22] the deepening of the surface mixed layer in the northern Red Sea during winter allows the transport of nutrients from the deep to the near-surface layers [20,23,24]. The variability of mixed layer depth (MLD) in the Red Sea is primarily controlled by the seasonally regulated ocean-atmosphere buoyancy fluxes [25–27]. During summer, warm temperatures lead to buoyancy gain (decreasing density), increasing stratification. Consequently, vertical mixing is suppressed across the entire basin leading to shallow mixed layers. In autumn, buoyancy loss (increasing density) starts to break the thermal stratification and initiates convective overturning and mixing, deepening the mixed layers throughout the entire basin [27,28]. The mixed layer spatial distribution presents a north to south gradient, generally reflecting the gradient of the atmospheric forcing, [29] which is stronger in the northern parts of the basin during wintertime and thus develop the deepest mixed layers [28], providing nutrients to the surface layers that ultimately increase CHL concentrations [16,18,20,24]. On the other hand, strong winds can also deepen the mixed layer through wind induced mixing, especially in the southern parts of the basin (see Fig 6 in Krokos et al. [27]). In spite of the atmospheric forcing being the dominant driver of MLD variability in the Red Sea, locally other processes such as advective fluxes and entrainment play an important role as well [28].

The influx of Gulf of Aden nutrient-rich waters, responsible for the mesotrophic levels of the southern basin [20], is regulated by seasonal atmospheric forcing and associated with the Indian monsoon [25,26]. Throughout the summer monsoon, the entire Red Sea experiences north-westerlies [29]. This wind pattern drives surface waters out of the Red Sea and into the Gulf of Aden. To counteract this mass loss, cooler, nutrient-rich waters from the Gulf of Aden, known as Gulf of Aden Intermediate Water (GAIW), intrude into the southern Red Sea at intermediate depths [25]. The pathway and mixing of GAIW with the ambient waters, including the productive coral reef banks [16], have been identified as the main contributing factors to the observed summer bloom in the southern regions of the basin [20,30,31]. However, the northward propagation of GAIW is interrupted by mesoscale features that occur in the central basin (between 18 to 24°N) [32,33], which alter the GAIW characteristics [34]. During the winter monsoon, a shift in wind direction to south-easterlies occurs in the southern half of the Red Sea. These monsoonal winds drive nutrient-rich Gulf of Aden Surface Waters (GASW) into the Red Sea [26] and are considered primarily responsible for the winter bloom in the south [18,20,35]. The GASW are further mixed in the central basin due to the intense mesoscale activity during winter [32] while their northward horizontal advection has been detected only up to 23ºN [36,37], leaving the northern Red Sea dependent solely on vertical mixing for nutrient supply.

Variations in vertical mixing alter the well-mixed surface layer and induce changes in both nutrient and light levels [38]. Enhanced thermal stratification of the water column due to global warming can lead to a shallower MLD, weakening the injection of nutrients into the euphotic zone and subsequently reducing phytoplankton biomass [39]. These trends have been evident through ocean colour observations in tropical regions over the past few decades [9,38,40,41]. The warm and oligotrophic nature of the Red Sea makes it particularly susceptible to the effects of warmer conditions. Recent studies have reported that the Arabian Peninsula experiences higher rates of warming compared to other regions [42], resulting in both physical changes [43–45], and biological responses in coral reef ecosystems [46–48] and in phytoplankton biomass [24,35]. Given this vulnerability, understanding the current control of MLD variability on nutrient distribution in the Red Sea basin is important. Although the mechanism of nutrient input through the mixed layer is well accepted, in the Red Sea it has been identified clearly only for the northern basin. The contribution of vertical mixing to surface CHL over the entire basin has not yet been investigated, leaving a gap in our understanding of the vertical advection of nutrients in the euphotic zone.

The scope of this study is to examine the co-variability between MLD and CHL over the entire Red Sea basin. By utilizing validated high-resolution spatiotemporal numerical MLD simulations and remotely-sensed CHL observations, we investigate regional differences of the MLD-CHL relationship at seasonal and interannual scales, aiming to provide valuable insights into the influence of vertical mixing on CHL concentrations in the Red Sea.

## Materials and methods

### Study area

**Fig 1:**
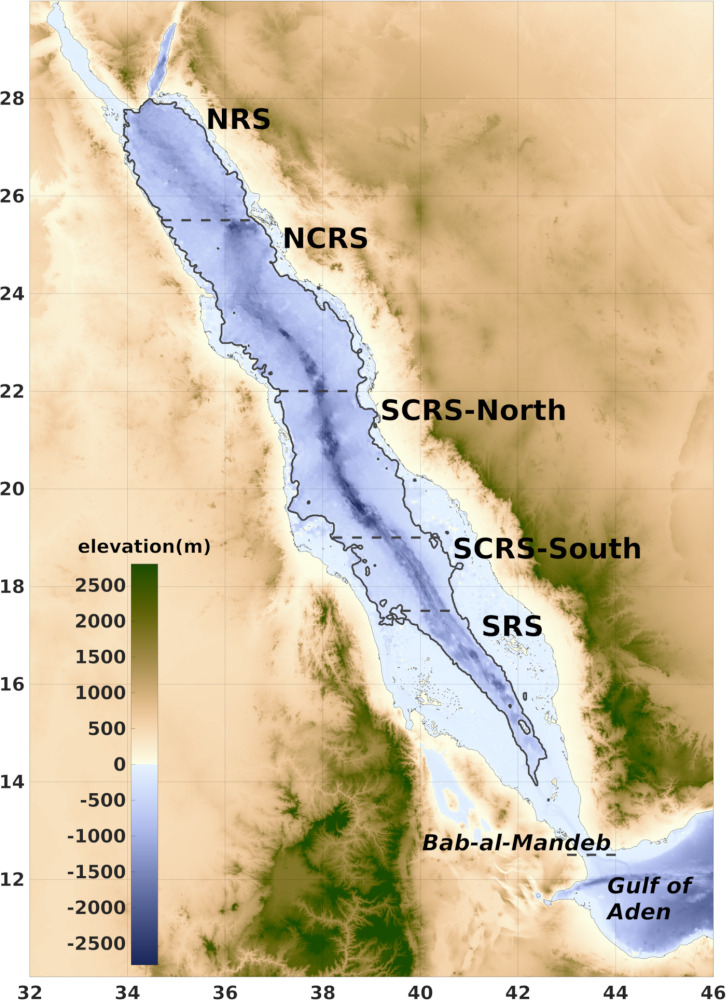
Bathymetry of the Red Sea and topography of Africa and Arabian Peninsula that surround the basin (GEBCO Compilation Group (2021) GEBCO 2021 Grid [ 49]**).** Black line denotes the 250 m isobath (determined from MITgcm bathymetric layers). Dashed lines set the boundaries of the sub-regions.

To investigate the relationship between MLD and CHL in the Red Sea our analysis is confined to the deep waters by the 250 m isobath, in order to eliminate the effects of the shallow banks with complex bathymetry and islands on the development of the mixed layer (Fig 1). The Gulf of Aqaba, due to its hydrographic separation, and Suez, due to its shallow bathymetry [50] are excluded from the present analysis.

The Red Sea was divided into subregions in order to capture the variability of the MLD-CHL relationship in areas with different hydrodynamic and environmental conditions. Following Raitsos et al. [20] we further isolated the Tokar Gap jet area in the South-Central Red Sea due to its unique characteristics, resulting in the sub-regions that hereafter will be named, from north to south, North Red Sea (NRS), North Central Red Sea (NCRS), South Central Red Sea-North (SCRS-N), South Central Red Sea-South (SCRS-S) and South Red Sea (SRS) (boundaries denoted with dashed line in Fig 1).

### Data

#### Satellite ocean colour data.

Satellite observational data are available on a broad spatiotemporal range, compared to *in situ* observations that are scarce in space and time. ESA’s Ocean Colour-Climate Change Initiative (OC-CCI) product (https://climate.esa.int/en/projects/ocean-colour/) is derived by merging four sensors (SeaWiFS, MODIS, MERIS, VIIRS) and available at daily temporal resolution. OC-CCI observations have been used extensively and proven suitable for the Red Sea [16,31,51–53]. In this study, we used a high resolution (1km), OC-CCI CHL dataset that has been tuned specifically for the Red Sea [51], and used for exploring the variability of CHL concentrations in coastal and open water regions [54–56]. The regional tuning was achieved by parameterising the standard algorithms used for retrieving chlorophyll-a concentration from reflectance data and validated against available in situ observations in the Red Sea [51]. The Red Sea ocean-colour model outperforms the standard algorithms, showing a bias close to zero (δ=−0.066 $mg/m^{3}$) and significantly lower Root Mean Square Error (Ψ=0.161 $mg/m^{3}$) (see Fig. 7 in Brewin et al. [51]).

Since OC-CCI is a merged product of satellites with different start and end times, the present study focuses on the years 2002–2013. This period was chosen because the overlap of at least two sensors (for 2002–2010: SeaWiFS, MERIS, MODIS, for 2011: MERIS, MODIS, for 2012: MERIS, MODIS, VIIRS, and for 2013: MODIS, VIIRS) offers the best possible coverage, while also avoiding the years after 2013 when decay in MODIS calibration was observed (http://dx.doi.org/10.5285/9c334fbe6d424a708cf3c4cf0c6a53f5). In the Red Sea, especially in the southern part during the summer monsoon, coverage is heavily affected by persistent clouds, water vapour, haze, sun-glint [57] and frequent dust storms [15] that affect the atmospheric correction and the retrieval of CHL from satellite [51]. To minimise the presence of missing data and avoid bias while spatially averaging, we constructed spatial composites of 4 consecutive days and removed any individual pixels where CHL concentration exceeded 10 mg/m^3^. The 4-day temporal resolution, besides improving coverage, accommodates a valid representation of phytoplankton growth typical timescale (less than one week) [58,59]. Moreover, log-transformation was applied to the 4-day CHL spatial dataset (as suggested by the OC-CCI manual), to introduce normality and remove the effects of extreme values [60].

#### Hydrodynamic model outputs.

Outputs from the latest regional simulation of the Massachusetts Institute of Technology ocean general circulation model (MITgcm) configured for the Red Sea at a resolution of 1 km (1/100°) horizontal grid and 50 vertical layers (z-coordinates) with an exponentially increasing thickness and a total of 18 layers in the first 150 m [27], were used in the present study. The simulation is able to resolve hydrodynamic processes extending from overturning circulation [25,26] to mesoscale processes [32]. The model’s forcing at its open boundary, including temperature, salinity, sea surface height and velocity conditions, are derived from GLORYS2 (version 4), the most recent ocean reanalysis product from Mercator Ocean (http://marine.copernicus.eu/). The model’s atmospheric forcing at its surface boundary are high-resolution (5 km) atmospheric fields, downscaled by the ERAInterim reanalysis [61] using the Advanced Research version of the Weather Research and Forecasting (WRF) model and assimilating available in situ and satellite observations of the region (e.g., [29,62,63]). The model’s configuration is further described in detail in Krokos et al. [27] and in section S1 of their respective supplementary material. The model’s outputs of potential temperature, salinity and potential density have been extensively validated against available observations in the Red Sea (CTD profiles), while sea surface temperature and sea level anomalies have been validated against satellite-derived observations (the model’s extensive validation is described in detail in Krokos et al. [27] and in section S3 of their respective supplementary material).

The MLD outputs used in the present study originate from the study of Krokos et al. [27]. MLD was calculated using the relative variance method [64] and validated against MLD estimates derived from CTD observations (see Fig. 3 in Krokos et al. [27]). The model reproduces well the seasonal variability of the upper layer stratification across the basin and the Root Mean Square Difference between observed and modelled MLD is 13.8 m [27]. Daily MLD estimates were linearly interpolated to the CHL satellite grid and temporally averaged over four consecutive days to match the CHL temporal resolution.

**Fig 2:**
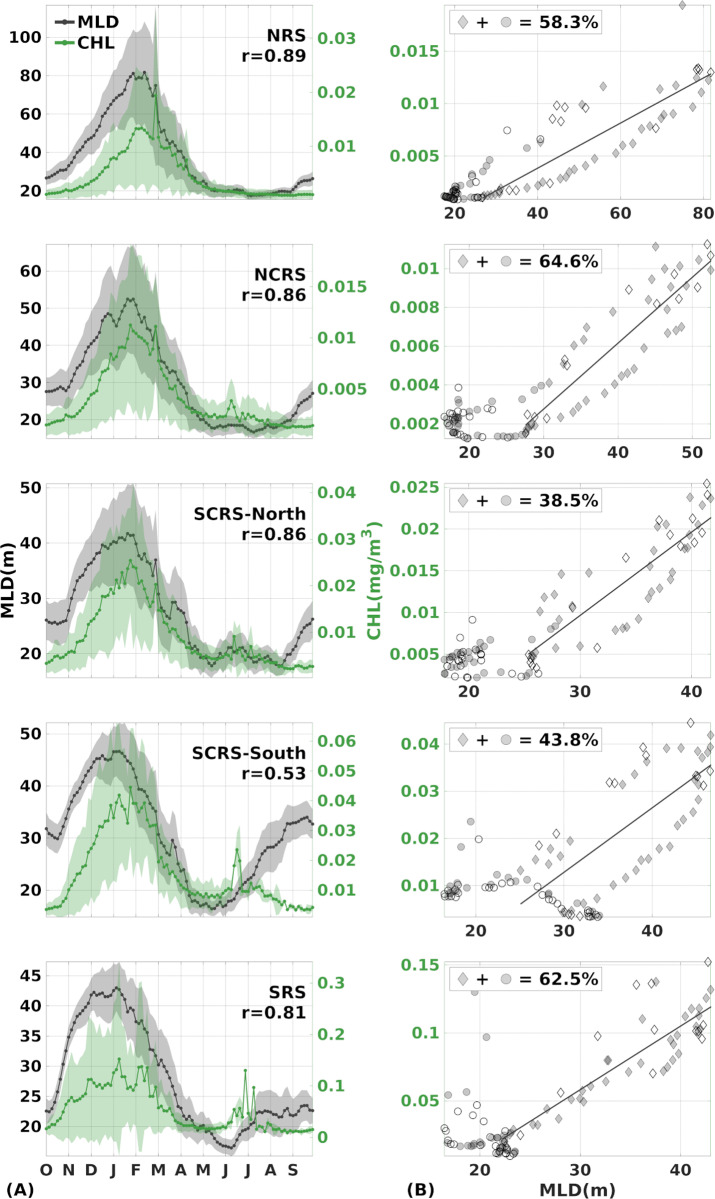
Annual cycle of MLD (grey) and CHL (green) 4-day composites of the Red Sea sub-regions. (A) Climatological seasonal cycle of average ±  standard deviation of MLD (grey) and CHL (green line) composites with their corresponding Pearson correlation coefficients at the 5% significance level (n = 96, p < .000) for each sub-region for 2002–2013 and (B) scatter plots of MLD vs. CHL 4-day seasonal cycle composites. Note that the diamond marking represents the winter, and the circle the summer. Filled data points indicate composites that their relative changes share the same sign (co-vary), accompanied by their percentage over the whole seasonal cycle. The grey line represents the fitted linear regression model during the winter (S1 Table).

### Analysis

#### Annual cycle.

Co-variability was examined first by analysing the seasonality of MLD and CHL concentrations. We created 4-day climatological maps of both variables by pixel-wise temporal averaging. Then, by spatially averaging each sub-region, the seasonal cycle of MLD was produced at a 4-day temporal resolution, while the seasonal cycle of CHL was produced after back-log-transformation. Pearson correlation coefficient at a 5% significance level (p < 0.05) was calculated for every cycle.

Corresponding average responses of CHL to MLD were evaluated from the relative changes between consecutive 4-day composites of the seasonal cycle. For variable X (MLD and CHL) at time t, the percentage changes were calculated as: $\Delta {Χ}_{rel}=\left(X_{t+1}-X_{t}\right)/X_{t}\cdot 100$.

The resulting values represent the relative changes of each variable with respect to its previous value, expressed as a percentage. Positive (negative) signs of the percentage changes indicate an increase (decrease) in the corresponding variable. Concurrent changes between MLD and CHL were determined when a positive (negative) change in CHL coincided with a positive (negative) change in MLD. An estimate of the co-variability can thus be expressed as a percentage of the total seasonal cycle composites where the signs of the percentage changes are equal: $sgn\left(\Delta MLD_{rel_{t}}\right)=sgn\left(\Delta CHL_{rel_{t}}\right)$.

Linear regression analysis provided further information about the relationship between MLD and CHL. The direction of the relationship was assessed through the fitted model, using MLD seasonal cycle composites as the independent variable and CHL as the dependent. A positive relationship is represented by a rising regression line while an inverse one by a falling regression line. The statistical significance of the model was evaluated through an F-test. Furthermore, the strength of the model was indicated by the coefficient of determination (R-squared) estimates the goodness-of-fit of the model. R-squared represents the proportion of the variance in the dependent variable that is predictable from the independent variable. It ranges between 0 and 1, where 0 indicates no relationship and 1 represents a perfect fit.

### Anomaly analysis

MLD and CHL anomalies were calculated as well to investigate their relationship, while standardisation allows a direct comparison between the datasets. The 4-day standardised anomaly spatial composites are estimated per pixel relative to the monthly climatology and standardised with the monthly climatological standard deviation using the formula: $Xanom_{t_{4day}}=\left(X_{t_{4day}}-X_{t_{month}}\right)/STD_{t_{month}}$.

We used monthly, instead of the long term, mean for calculating standardised anomalies in order to remove the seasonal component, while the baseline time series of 10 years is sufficient for calculating monthly means as a reference condition.

Anomaly correlation maps are useful for quantitatively exploring relationships between two variables. Spatial distribution of Pearson correlation coefficients r was calculated per pixel, omitting gaps using the 4-day spatial composites. To test the statistical significance of r at the 95% significance level, the p-value was calculated per pixel, disregarding gaps, $p=2\cdot \left[1-tcdf\left(\left|t\right|,n-2\right)\right]$, using a t-distribution, $t=r\cdot \sqrt{n-2}/\sqrt{1-r^{2}}$, with n the sample size.

The 4-day composites ensure coverage of at least 40% per pixel which provides a sample size large enough (n ≥ 100 out of the total N = 253), to calculate the correlations that are significantly different from zero (p < 0.05).

To further examine the relationship interannually, we created anomaly timeseries by spatially averaging the 4-day standardised anomaly spatial composites per sub-region. Prior to the spatial averaging, every sub-region that displayed spatial coverage in CHL less than 10% at a 4-day composite level was extracted to ensure robust spatial averaging. Then, the Pearson correlation coefficient (r) at a 5% significance level was calculated for every sub-region for the 10-year time series and on a seasonal temporal resolution (three months) as well.

To investigate the relationship between extreme anomalous MLD on surface CHL concentration, we adapted the well-established algorithm introduced by Hobday et al. [65], originally developed for detecting marine heatwaves (MHWs) to detect abnormally deep or shallow mixed layers. The methodology involves using a seasonally varying climatology to determine if the values of the considered variable during a specific period were exceptionally high or low. The daily climatology and percentage threshold were calculated by averaging an 11-day window around each daily climatology and further smoothed using a 30-day moving window mean. The intensity threshold for the high extremes was defined as the values in the daily MLD timeseries surpassing the 90th percentile of recorded climatology, while for the low extremes, the daily values below the 10th percentile. To qualify as a mixed layer extreme event that could significantly impact CHL in the Red Sea, these conditions had to persist for at least four consecutive days. The adjustments in percentile thresholds and minimum duration of extremes were made in alignment with the approaches of Hobday et al., [65] and the Special Report on the Ocean and Cryosphere in a Changing Climate (SROCC) of the Intergovernmental Panel on Climate Change (IPCC) [66], considering the specific scientific question and region. To determine the initiation/termination dates of the events we utilised the MATLAB toolbox M_MHW [67]. The toolbox can be accessed at https://github.com/ZijieZhaoMMHW/m_mhw1.0. After the dates of MLD extremes were determined, the response in the daily CHL anomaly time series was estimated as a percentage of all the MLD extremes where, $\left|CHL_{anom}\right|>0.5.$

Finally, to demonstrate the anomalous MLDs that sustain high CHL or lower surface CHL, we identified the co-occurrence of both MLD and CHL extreme events. Such simultaneous or closely successive occurrences can be considered as “compound events” [68,69], because the interaction of deeper/shallower MLD and higher/lower CHL can have a synergistic effect on marine ecosystems. For instance, an abnormally deep MLD may bring nutrient-rich water to the surface, enhancing CHL concentrations, while a shallow MLD might limit nutrient availability, reducing CHL levels. Such an approach has been already used to understand the combined effects of multiple stressors on the marine environment, including factors such as temperature, precipitation, hydrogen ion concentration, and CHL concentration [e.g., 70–72]. After applying the aforementioned methodology to the daily CHL timeseries, with the percentile conditions lasting for at least three consecutive days, we identified the dates of the compound events when extremely deep (shallow) mixed layers overlapped with extremely high (low) CHL. Given the significance of data coverage in our analysis, gap-free periods were selected to allow an accurate visual representation of compound events after spatially averaging the corresponding daily standardised anomaly maps over their duration period.

## Results

To assess the relationship between MLD and CHL, first we described the co-variability of the annual cycle in every sub-region of the Red Sea. We then focused on the winter period, where the relationship was stronger, and examined the spatial patterns and interannual variability of anomalies. Lastly, we presented indicative examples where simultaneous occurrences of extreme anomalies in the two variables took place.

### Seasonal co-variability of MLD and CHL

Annually, the variables exhibited a strong positive correlation in every region, with the NRS showing the deepest MLDs and the SRS displaying the highest CHL concentrations (Fig 2A). Across the entire basin, both MLD and CHL gradually increased in parallel from October, reaching their peaks in January to early February, and sustained high values until March.

From April to mid-May, both variables gradually decreased, and from May onwards, the subregions exhibited distinct characteristics. In the NRS, the mixed layer continued to shoal, reaching its lowest values in July - August, while CHL remained consistently low throughout the entire summer. The same applied to the NCRS and SCRS-N, with a noticeable period in June, characterised by smaller peaks in both MLD and CHL. In the SRS, the shoaling of the mixed layer continued until mid-June, after which deepening started. The SCRS-S, however, exhibited a steep deepening that continued until September, presenting the deepest mixed layer observed in the entire basin during the summer months. In the SRS, mixed layer depths deepened until July, before stabilising at a deeper plateau. CHL concentrations in the south remained low until the beginning of June, followed by the summer peak. The summer bloom lasted for almost two weeks in the SCRS-S and extended for one month in the SRS, with a much higher magnitude. After these mid-summer peaks, CHL concentrations dropped again. September marked the initiation of mixed layer deepening throughout the basin, but CHL values remained low during this month. Consequently, we define the broader winter blooming period lasting from October to March and the summer period from April to September.

On average, the two variables displayed a synchronous evolution in 60% of the annual cycle’s composites (Fig 2B, see filled data points). That is, when MLD was increasing (decreasing) between two consecutive composites, CHL was also increasing (decreasing) accordingly. A relative increase of 3% in MLD coincided with an average CHL increase of 5% and 13% in the northern and southern basins, respectively (Table 1). Conversely, when MLD shoaled by 4.5% on average, there was a corresponding 5.6% decrease in CHL in the whole basin, except for the SRS where a 9% relative decrease in CHL occurred when MLD became shallower by 3%. Evidently, CHL in the southern basin, especially in the SRS, showed considerable changes even with small MLD variations.

**Table 1:** Relative positive (negative) change between consecutive MLD climatological 4-day composites appearing with simultaneous positive (negative) relative change in the CHL climatological 4-day composites in the five Red Sea sub-regions. The relative changes of each variable are calculated with respect to their previous value and expressed as a percentage.

	MLD	CHL	MLD	CHL
	increasing by (%)		decreasing by (%)	
**NRS**	3.5	4.3	‒5.5	‒5.3
**NCRS**	3.1	4.7	‒4.5	‒6.7
**SCRS-N**	3.3	5.7	‒3.8	‒5.6
**SCRS-S**	2.9	10.0	‒4.4	‒4.8
**SRS**	2.9	16.9	‒3.1	‒9.0

The seasonality was further investigated by fitting a linear regression model to the two seasons separately (Fig 2B). During the winter season, the slope of the linear model was found to be positive in every sub-region of the Red Sea, indicating that CHL increases as MLD increases. Notably, at least 70% of CHL’s variance could be explained by MLD (R-squared >  0.7), in the whole basin, except for the SCRS-S (R-squared =  0.5) (S1 Table). In contrast, during the summer period, R-squared remained high only for the NRS, while for the rest of the basin, it was close to zero (S1 Table). This result indicates that CHL’s variance is not well explained by the shallow, summer mixed layers.

**Fig 3:**
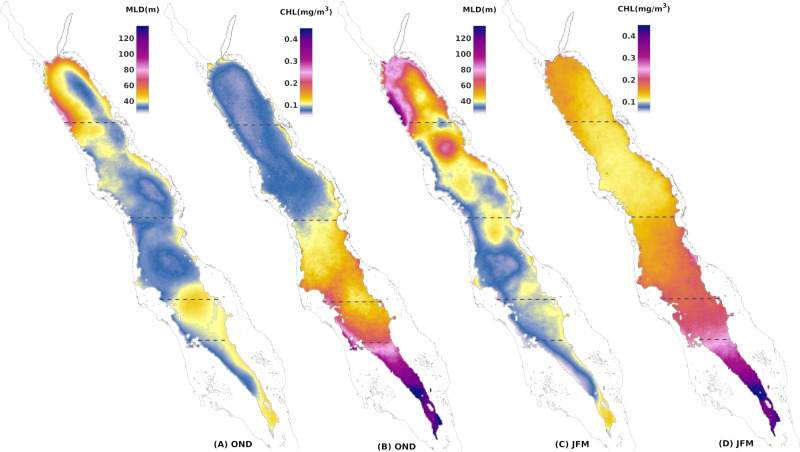
Seasonal climatological fields of mean MLD(m) and median CHL(mg/m^3^) for OND (A and B) and JFM (C and D) in the Red Sea. Dashed lines set the boundaries of the sub-regions.

Given the low variability of MLD during the summer season and the inability to establish a clear relationship with CHL, we focused on the broader winter blooming period that was further divided into two seasons to depict the processes more accurately. October to December (OND) represents the onset of mixed layer deepening and increasing CHL concentrations, while January to March (JFM) represents the peaks of both variables and the onset of mixed layer shoaling and decreasing CHL concentrations (Fig 3).

By analysing MLD and CHL standardised anomalies, after removing the seasonal cycle, we can better isolate and understand the deviations from the typical seasonal patterns during the critical winter blooming period in the Red Sea.

### Spatial correlations between CHL and MLD anomalies

**Fig 4:**
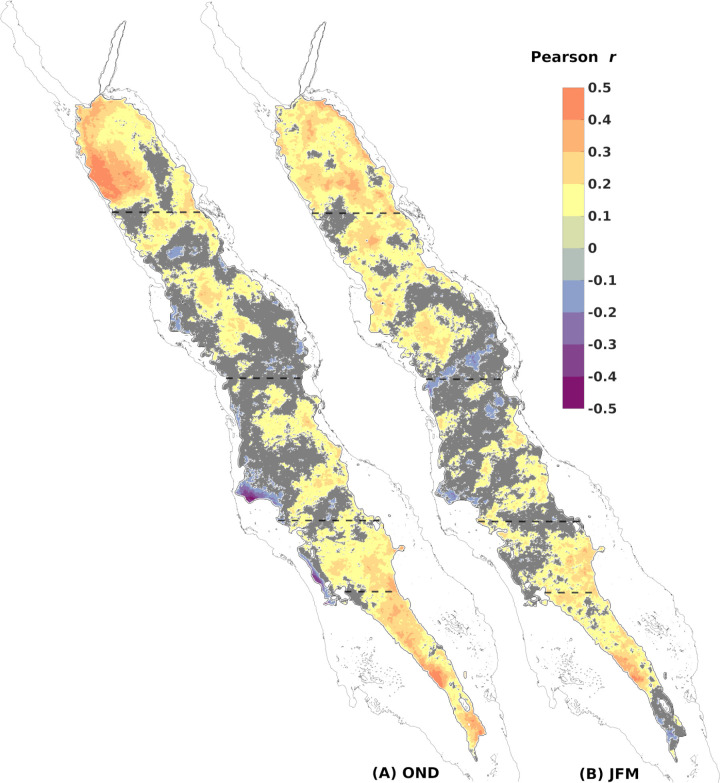
Maps of Pearson correlation coefficients between MLD and CHL standardised anomaly 4-day composites. (A) October-November-December for 2002–2012 and (B) January-February-March for 2003–2013 (significance level 5%, n: at least 100 out of the total **n** =  253). Orange colour scale depicts increasing positive correlation, purple depicts negative and grey colour marks insignificant correlations, where p > .05). Dashed lines set the boundaries of the sub-regions.

To identify spatial dependencies between MLD and CHL anomalies, we computed climatological correlation coefficient maps for the two winter seasons (OND and JFM). The resulting spatial distribution (Fig 4) unveiled diverse patterns. In the northern and southernmost regions of the basin, MLD and CHL anomalies showed a strong positive correlation. However, in the central basin, the positive correlation was weaker and inhomogeneous.

At the onset of winter (OND) (Fig 4A), the SRS and the eastern SCRS-S exhibited strong positive correlations, reaching values of up to ~ 0.4 in localised regions. Moving northwards, the central Red Sea showed a predominantly patchy distribution of weak correlations, surrounded by areas with insignificant values. Notably, negative correlation values emerged locally in the northern part of the NCRS. In the far north of the basin, the NRS displayed spatially homogeneous, strong, and positive correlations, especially in the western part, with values reaching up to 0.5 – the highest observed throughout the basin. The NRS during JFM was still occupied by positive correlations, particularly along the eastern boundary, although weaker and more irregular compared to the OND period. On the other hand, the NCRS and SCRS-N were still dominated by insignificant correlation values. In the eastern SCRS-S and most of the SRS, MLD and CHL anomalies remained positively correlated during JFM, although less homogenous than in early winter.

### Interannual co-variability of MLD and Chl

#### Time series of MLD and CHL anomalies.

To gain a deeper understanding of the relationship revealed by the correlation maps of MLD and CHL anomalies, we further investigated the significance of the relationship on the MLD and CHL anomaly 4-day time series. Positive (negative) anomalies indicate deeper (shallower) MLD and higher (lower) CHL concentrations compared to their corresponding monthly climatologies.

**Fig 5:**
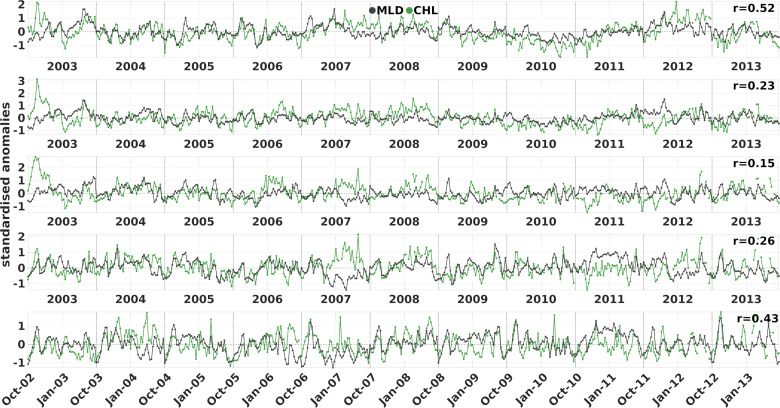
Time series of MLD (grey) and CHL (green) standardized anomaly 4-day composites in the five Red Sea sub-regions. The time series present the months October to March from 2002 to 2013 for each sub-region with their corresponding Pearson correlation coefficients.

We examined the lagged Pearson correlation coefficient between the time series within the first 10 lags (i.e., 40 days) at the 5% significance level. Every region displayed a positive correlation value, with the highest coefficients observed at lag zero (Fig 5). Specifically, the NRS and SRS exhibited the highest correlation values (r =  0.52; 0.43 respectively, n =  498; 488, p < .000). In the central Red Sea, MLD and CHL were weakly related (r =  0.23; 0.15; 0.26 for the NCRS, SCRS-N and SCRS-S respectively, n =  499; 182; 488, p < .000). However, it is important to note that a single correlation value for the entire time series underestimates the true relationship. The seasonal (3-month) correlation coefficients (S1 Fig) show that the relationship between MLD and CHL is much stronger during certain winters. For example, during JFM of 2008 and 2012 correlations in all three sub-regions of the central Red Sea reach up to r ≈  0.7 (p > .00), while the presence of weakly and insignificantly correlated seasonal intervals, such as JFM 2007 and 2011, can influence the overall correlation estimate.

#### Co-occurrences of anomalous events.

The correlation between MLD and CHL anomaly time series indicated a positive relationship across the Red Sea, though with varying strength and under the presence of considerable variability throughout the 10 years*.* To focus on the most extreme deep or shallow MLDs over shorter time scales, we lastly utilised the anomaly detection algorithm originally developed by Hobday et al., [65] for detecting marine heatwaves (MHWs) on the daily MLD time series. Thus, we identified anomalous MLD events that last for a minimum of four consecutive days*.* After examining the CHL anomalies during these MLD extremes, we also applied Hobday’s algorithm on the daily CHL time series, to detect specific cases of CHL extremes and identify potential cases of deep MLD – high CHL, or shallow MLD – low CHL compound events.

Several anomalous MLD periods were detected in each region. In 50% of the NRS’s extreme days, CHL standardised anomalies exceeded 0.5 standard deviations above the mean. In the NCRS, the percentage drops to almost 20%, while in the central basin (SCRS-N and SCRS-S), the percentage is even lower (10%). Extreme deepening in the SRS results in CHL anomalies above half standard deviation in 30% of days of occurrence, while extreme shoaling leads to anomalies below ‒0.5 standard deviation in 20% of the shallow MLD extreme days (S2 Table).

**Fig 6:**
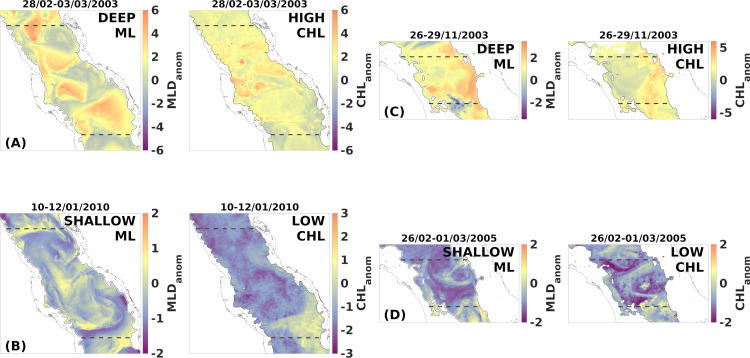
Temporal averages of daily standardised anomaly maps during compound events of MLD (left column) and CHL (right column). (A) Positive compound event (deep ML/ high CHL) and (B) negative compound event (shallow ML/ low CHL) in the NCRS respectively, while (C) and (D) positive and negative compound events in the SCRS-S respectively.

Here, we present four indicative cases of compound events from the central basin (NCRS and SCRS-S), that highlight notable associations. Anomalously shallower MLD periods over both sub-regions (Figs 6B and 6D) correspond to anomalously widespread low surface CHL concentration. Extreme high surface CHL overlaps with deep mixed layers in the NCRS (Fig 6A) from the 28th of February until the 3rd of March, 2003. Besides the obvious overall deepening, mesoscale activity is also anomalously high during this time interval as implied by the spatial distribution of MLD anomalies that locally reach even up to 6 standard deviations above the monthly climatology. In the SCRS-S, a period with anomalously positive MLD anomalies, with the highest deepening observed in the eastern part of the basin, spatially overlaps with very high CHL concentrations, reaching up to 5 standard deviations above the November climatology (Fig 6C).

## Discussion

This study investigated the relationship between modelled MLD and satellite-derived CHL concentrations in the oligotrophic Red Sea. The contribution of vertical mixing to surface CHL concentration is influenced by the strong seasonality and variability of the regional processes over the five considered sub-regions of the Red Sea (Fig 1). In the entire basin, the annual cycles of MLD and CHL are significantly related (r > 0.8, p < 0.00), except for the SCRS-S where the correlation is weak (r = 0.53, p < 0.00) (Fig 2A). On average, 60% of CHL’s annual cycle composites co-vary with MLD in every sub-region of the basin (Fig 2B, see filled data points), highlighting the significant role of vertical mixing in surface CHL variability in different regimes of the basin, from the far oligotrophic northern basin to the mesotrophic southern areas (Figs 3B and 3D).

At the onset of winter, intense buoyancy loss and wind-driven turbulence weaken stratification and induce convective mixing, with a consequent deepening of the mixed layers throughout the Red Sea [27]. Thus, vertical mixing provides nutrients to the surface layer, increases surface CHL concentration and marks the initiation of the blooming period. Over the basin, both CHL and MLD reach their peaks in January-February before the onset of summer thermal restratification. As the surface layer becomes more stratified, vertical movements are suppressed and the mixed layer shoals, depleting the surface of nutrients and ultimately decreasing surface CHL concentration (Fig 2A). Linear regression analysis further revealed that CHL variability can be significantly predicted by MLD during winter, when approximately 70% of CHL’s variance was explained by the fitted MLD data throughout the basin, except from the SCRS-S where the respective explained variance was ~ 50% (S1 Table).

Co-variability of MLD and CHL during the summer was very weak since shallow mixed layers and low CHL values persist throughout the summer everywhere in the whole basin (Fig 2A). This result is in agreement with the expected effect of thermal stratification on surface nutrient depletion and consistent with previous work [18]. However, some short-term features of elevated CHL and slightly increased MLD are revealed by the climatology during the warm stratified period. For instance, spatially averaged MLD and CHL in the NCRS and SCRS-N showed a small increase during June (Fig 2A). Dynamical features, such as eddies, present in the northern and central basin during summer have been reported to advect coastal nutrient-rich waters to the open sea [15–17,20] while seemingly deepening the spatial average of MLD. Therefore, the observed peaks in both variables are unrelated to nutrient enrichment from deeper mixed layers and can be attributed to horizontal advection through mesoscale features. Despite vertical mixing being confined to a shallow surface layer, the SRS exhibits enhanced surface CHL concentrations from June to July, extending to the SCRS-S province (Fig 2A). This feature has been reported in several previous studies and has been attributed to the upwelling and dispersion of the GAIW [30,31] along the eastern shores [73]. Interestingly, the MLD seasonal cycle in the SCRS-S, displays an inverse relationship with CHL from July to September, after the short summer peak (Fig 2A). In this region, located around 19°N, a semi-permanent anticyclone is developed during July, driven by the Tokar Gap wind jet [74]. Anticyclonic eddies rotate clockwise in the northern hemisphere and downwell water to deeper layers, causing the stretching of isopycnals and reducing nutrient and CHL levels at its surface [75]. The impact of anticyclonic gyres on surface CHL in the Red Sea has been reported through satellite [15] and *in situ* [37] data. Thus, the deep mixed layers attributed to downwelling along with the low CHL values observed in the SCRS-S climatology from July to September, depict the semi-permanent Tokar anticyclonic eddy that deepens locally the mixed layer [28] by suppressing the isopycnals, leaving the surface depleted of nutrients. Because thermal stratification minimises MLD variations in summer, and the observed CHL summer peaks are unrelated to the transfer of nutrients through vertical mixing, the rest of our analysis was focused on the winter period, when strong linear relationships were observed in the annual cycle and linear regression analysis.

During winter, the reversal of winds in the southern half of the Red Sea changes the direction of the surface water flow, redirecting it into the Red Sea from the Gulf of Aden. The nutrient-rich Gulf of Aden Surface Water (GASW) has been regarded until now as the primary factor contributing to the mesotrophic levels of the southern basin, along with the advection of nutrient-rich waters from the productive coastal reefs. While these advective processes have been reported [15,18,20,35], here, we showed that the seasonal cycles of MLD and surface CHL are strongly correlated in the SRS. The significant positive correlation of standardised anomalies observed in most of the SRS spatially (Figs 4A and 4B) and interannually (Fig 5), suggests that surface CHL is not only affected by the winter intrusion of nutrient-rich surface waters, but it is closely linked to MLD anomalies as well. Krokos et al. [28] has shown that in the SRS during the autumn deepening, cooling occurs at the base of the mixed layer. This cooling depicts the entrainment of cooler and fresher GAIW that have accumulated at intermediate depths towards the end of the summer period. These remnants of GAIW have been observed in the central basin until November [30,76,77]. Deepening of the mixed layer in the SRS is thus crucial for redistributing nutrient-rich water masses from the intermediate layers of the GAIW intrusion. This is further confirmed by the relative changes between consecutive seasonal composites, showing that even a slight change (3%) in the SRS’s MLD, displays one of the biggest responses in surface CHL observed in the basin (10%) (Table 1).

In the SCRS-S, the impact of vertical mixing to surface CHL can be detected from the high and homogenous positive spatial correlations at the eastern part of the basin (Figs 4A and 4B), where the deepest mixed layers are formed due to the prevailing south-easterlies channelling through Bab-el-Mandeb Strait (Figs 3A and 3C). Although the high interannual variability lowers considerably the correlation of the anomaly time series, the relationship is actually underrated by one value. As can be seen from the correlation coefficients at a shorter temporal scale, individual winters, such as JFM 2009, display a much stronger correlation (r =  0.9, p < .000) (S1 Fig). This can be demonstrated as well by a compound event of anomalous deep mixed layer in the SCRS-S that overlaps spatially with exceptionally high CHL concentrations, particularly in the eastern part of the sub-region (Fig 6C), or an event of extremely shallow mixed layer that is in agreement with a CHL-depleted period (Fig 6D).

The central Red Sea during winter displays the weakest correlations observed spatiotemporally, indicating that vertical mixing is not the primary driver of surface CHL. The spatial distribution of the climatological anomaly correlation values in the SCRS-N and NCRS (Fig 4) are non-uniform, mostly insignificant and weakly positive or even negative in the northern part of the NCRS. The circulation over the central Red Sea (18‒24 °N) is characterised by intense mesoscale activity [32,33] and thus, surface currents are expected to transport nutrients and CHL. Thus, correlation values between MLD and CHL are not expected to be homogenous, as confirmed by our results. Mesoscale eddies have been reported to transport nutrients and ultimately regulate phytoplankton biomass [75,78]. In the Red Sea, Raitsos et al. [17] used CHL ocean colour images to trace circulation pathways between the east and west coasts of the Red Sea, revealing the significant role of surface currents and eddies in horizontally-transferring nutrient and/or CHL rich waters from the productive coral reef ecosystems [79]. Moreover, sub-mesoscale currents or fronts can influence phytoplankton concentration [80], features that have also been reported in the central Red Sea [79]. Through *in situ* observations, Zarokanellos et al. [76] demonstrated the contribution of eddies in the northward transport of GASW and identified eddy-related horizontal advection as the primary mechanism for nutrient transport in the central Red Sea. Acker et al. [15] reported cases of mesoscale features in the central Red Sea influencing surface CHL concentration detected by satellites, where anticyclonic and cyclonic eddies were identified from the low and high CHL concentration at their centres, respectively. Fig 4A offers an example of the role of an anticyclonic gyre in surface CHL at approximately 25°N. This semi-permanent anticyclone that is characterised by deeper mixed layers [28,32], is accurately depicted by negative anomaly correlation, assuming low CHL concentration at its centre due to downwelling. The peripheral insignificant values can be justified by the variable position and radius of this dynamical eddy. In spite of the overall weak correlation depicted in the correlation of the anomaly timeseries as well (Fig 5), occasional contribution of deep mixing to surface CHL in the central basin has been reported through *in situ* data [81], which can still be detected in our results over shorter time scales. For instance, during compound events, a generalised CHL response matches the anomalous mixed layers (Figs 6A and 6B), whereas during specific periods, the seasonal correlation values of the anomaly time series can reach r = 0.7 (p < .00) (S1 Fig).

Lastly, in the NRS vertical mixing is highly related to CHL since the northern parts of the basin develop the deepest mixed layers (Figs 3A and 3C) due to intense buoyancy loss during winter [27,28]. Previous studies at a shorter temporal resolution have shown that nutrient redistribution from deeper layers through winter convective mixing dominates phytoplankton biomass in the NRS, as evidenced by satellite [16,20,24,82] and *in situ* data [23,54,55]. Our analysis confirms these findings, as the NRS exhibits the highest correlation in the seasonal cycle (0.89) and MLD variability explains at least 70% of CHL’s variability during the whole year. Interannually, the relationship of the anomalies is the strongest (r =  0.52, p < .000), while the highest spatial correlation of anomalies in the whole Red Sea is observed in the western NRS during OND (Fig 4A), coinciding with the area of maximum wind stress [83]. Interestingly, during JFM, when the deepest mixed layers have formed (Fig 3C), the spatial distribution of anomaly correlation values are weaker (Fig 4B), implying that surface CHL responds to a greater extent to anomalous mixed layers after the summer depletion of nutrients, at the initiation of active deepening and blooming, rather than during the period that covers the winter bloom and the deepest mixed layers. Indeed, extreme deep convective mixing can either reduce light exposure, decreasing phytoplankton growth or diluting phytoplankton biomass vertically and decreasing CHL concentrations at surface [84]. When MLD extends beyond the depth of the euphotic layer (Ze), it can impose light limitation to phytoplankton [85] or possibly dilute grazers [86]. Indeed, decreasing phytoplankton surface net growth during a year of extreme deep mixing has been reported using ecosystem modelling in the Gulf of Aqaba [87] and in the Arabian Sea [88]. Light availability could be a limiting factor for phytoplankton in the NRS during JFM as well, since MLD reaches and exceeds the euphotic depth (S2 Fig), which explains the weaker positive relationship we observe at the peak of the deepening. Further investigation, incorporating the vertical distribution of CHL through *in situ* data and ecosystem modelling, is needed to confirm this observation.

Since CHL’s vertical distribution is not accounted for in this analysis, limitations emerge from the hypothesis itself that vertical mixing (as indicated by MLD) transfers nutrients from the deeper layers to the surface triggering new production (as indexed by CHL). Although this is a widely accepted mechanism, the following have to be taken into consideration. First, we assume that the deep layers of the broader Red Sea are an important source of nutrients, although in situ datasets of nutrient availability throughout the water column are scarce. However, except for the SRS, which is impacted by the nutrient-rich waters from the Gulf of Aden, alternative nutrient sources aside from the deep layers (e.g., rivers, rainfall, dust storms, coral reefs etc.) are limited [12] and sporadic. Thus, vertical nutrient supply from the deeper layers is likely the remaining dominant source of nutrients for the rest of the basin. Secondly, we assume that the satellite-derived CHL accounts for new production, although we may overlook possible redistribution of CHL from the Deep Chlorophyll Maximum (DCM), a permanent feature in stratified tropical seas such as the Red Sea [89]. However, its depth lies at the end of the euphotic zone [54,90], which rarely exceeds the depth of the mixed layer (S2 Fig). In the NRS where the depth of the euphotic zone during winter can be deeper than the mixed layer (S2 Fig), in situ CHL concentration decreases with depth [54]. Indeed, Gittings et al. [54] reported that during the main winter growth period (Dec-Mar), characterized by high surface CHL, vertical profiles show the complete erosion of the DCM, with substantially higher and homogenous CHL concentrations in the upper mixed layer that decrease with depth. This indicates that since vertical mixing imposes homogenous conditions within the surface mixed layer during the winter, variations in surface CHL reflect new production rather than redistribution from the DCM.

## Conclusions

This study evaluated the relationship between vertical mixing, proxied by the depth of the mixed layer, and satellite-derived phytoplankton biomass in the whole Red Sea, a typical oligotrophic tropical sea with limited external nutrient input besides the nutrient-rich deep layers. Although in the northern Red Sea the mixed layer has been reported as the main driver of surface CHL concentrations, in the rest of the basin, its contribution had not been investigated comprehensively, with the exception of few *in situ* surveys and case-studies. The results presented here showed that the seasonal cycle of CHL co-varies significantly with MLD in every province of the Red Sea. Further spatiotemporal analysis at seasonal and interannual time scales revealed though that the relationship varies with latitude. CHL in the northern basin was highly associated to the mixed layer’s variability as expected, although the relationship was weaker during the peaking period of both variables, a contrasting result attributed to mixed layers deep enough to dilute CHL and nutrients away from the euphotic zone. In the central basin, phytoplankton biomass was weakly related to MLD and, as displayed in the spatial distribution of the correlation values, governed by the mesoscale surface flow, although occasionally extreme CHL events could be rooted back to MLD anomalies. The southern basin’s higher CHL concentration was until now almost solely been attributed to the horizontal advection of nutrient-rich surface waters from the Gulf of Aden. However, our analysis shows that MLD and CHL are strongly correlated in the south Red Sea, with even minor MLD variations leading to significant CHL changes in the southern basin, more than in any other region. This finding reveals the importance of vertical mixing in regulating the redistribution of the Gulf of Aden nutrient-rich waters at surface after they enter the basin.

Projections of decreasing CHL levels in tropical oceans have been partially attributed to the shoaling of the mixed layer by inhibiting nutrient injection to the euphotic layer. However, the regional impacts of warming on the ecosystem dynamics of the Red Sea region are not yet clear. Producing regional projections through operational biogeochemical models and on-going *in situ* monitoring is thus essential for the effective ecosystem management that will ensure the long-term sustainability of the basin.

## Supporting information

S1 Table
Linear regression results in the Red Sea sub-regions, using MLD seasonal cycle composites as the independent variable and CHL as the dependent.
Regression models, CHL=a⋅MLD+b, were applied to the winter (n =  48) and summer seasons (n = 48) separately (detailed results of the summer season were omitted, since the relationship was weak as indicated initially by the R-squared coefficient of determination).(DOCX)

S2 Table
Extreme MLD days with simultaneous $\left|CHL_{anom}\right|\geq 0.5$ , in the five Red Sea sub-regions.
Occurrences of extreme MLD days (deep or shallow), are detected by the MHW algorithm [65] and expressed as a percentage of the total daily timeseries (October 2002 to March 2013: 2005 days), along with the corresponding days where $\left|CHL_{anom}\right|\geq 0.5$, expressed as a percentage of the total extreme MLD days.(DOCX)

S1 Fig Timeseries of winter Pearson correlation coefficients between MLD and CHL standardized anomaly 4-day composites at 3-month intervals (OND and JFM) (5% significance level, n =  23) from 2002-2013 in the five Red Sea sub-regions.
Green indices mark the significant correlation values (p < .05) while black indices the insignificant (p > .05).
(TIF)

S2 Fig
(A) Monthly seasonal cycle (averaged from 2002-2013) of MLD (grey), CHL(green) and euphotic depth (Ze) (orange) for each sub-region of the Red Sea and spatial distribution of differences between seasonal spatial climatologies of Ze and MLD during (B) OND and (D) JFM.
The definition of the euphotic depth, $Z_{e}=\frac{4.605}{K_{d}(PAR)}$, [91] requires the calculation of K_d_(PAR), the diffuse attenuation coefficient of Photosynthetically Active Radiation (PAR). K_d_(PAR) represents the decay of the solar irradiance as it propagates into the water column and was calculated according to [92], using the formulas $K_{d}(PAR)=4.6051.K_{d}(490)/(6.07.K_{d}(490)+3.2)$, for K_d_(490) ≤ 0.115m^-1^ and, for K_d_(490) > 0.115m^-1^. K_d_(490), the diffuse attenuation coefficient at λ=490nm, was derived from satellite observations, available from ESA’s Ocean CCI product (https://climate.esa.int/en/projects/ocean-colour/).(TIF)
