# Supplementary material for: Unravelling the influence of mixed layer depth on chlorophyll-a dynamics in the Red Sea
Source: PLoS One. 2025 Mar 5;20(3):e0318214. doi: 10.1371/journal.pone.0318214 (PMC11882056; doi:10.1371/journal.pone.0318214)
Supplement: S1 Table — Regression models, CHL=a⋅MLD+b, were applied to the winter (n = 48) and summer seasons (n = 48) separately (detailed results of the summer season were omitted, since the relationship was weak as indicated initially by the R-squared coefficient of determination). (DOCX) [file pone.0318214.s001.docx]

|  | **Summer** | **Winter** | | | | | | |
| --- | --- | --- | --- | --- | --- | --- | --- | --- |
|  | **R-squared** | **R-squared** | **F-stat**  **(DF=46)** | **p-value** | **a** | **p-value** | **b** | **p-value** |
| **NRS** | 0.691 | 0.7147 | 115.22 | 4.076e-14 | 0.0002154 | 4.076e-14 | -0.0047772 | 0.000121 |
| **NCRS** | 0.0214 | 0.8003 | 184.39 | 1.05e-17 | 0.0003394 | 1.05e-17 | -0.0074202 | 2.58e-09 |
| **SCRS-N** | 0.0328 | 0.7522 | 139.66 | 1.549e-15 | 0.0009967 | 1.549e-15 | -0.020251 | 9.44e-09 |
| **SCRS-S** | 0.3366 | 0.5339 | 52.685 | 3.738e-09 | 0.0013664 | 3.738e-09 | -0.028167 | 0.000321 |
| **SRS** | 0.0852 | 0.7555 | 142.11 | 1.143e-15 | 0.0046826 | 1.143e-15 | -0.082239 | 4.44e-07 |
