# Supplementary material for: Unravelling the influence of mixed layer depth on chlorophyll-a dynamics in the Red Sea
Source: PLoS One. 2025 Mar 5;20(3):e0318214. doi: 10.1371/journal.pone.0318214 (PMC11882056; doi:10.1371/journal.pone.0318214)
Supplement: S2 Table — Occurrences of extreme MLD days (deep or shallow), are detected by the MHW algorithm [65] and expressed as a percentage of the total daily timeseries (October 2002 to March 2013: 2005 days), along with the corresponding days where CHLanom≥0.5, expressed as a percentage of the total extreme MLD days. (DOCX) [file pone.0318214.s002.docx]

|  | **Deep MLD** (% of 2005 days) | **CHL_anom_≥0.5** (% of the deep MLDs) | **Shallow MLD** (% of 2005 days) | **CHL_anom_≤-0.5** (% of the shallow MLDs) |
| --- | --- | --- | --- | --- |
| **NRS** | 8 | 45 | 8 | 48.4 |
| **NCRS** | 9.1 | 19.1 | 8.4 | 28 |
| **SCRS-N** | 8.9 | 13.5 | 8.4 | 11.2 |
| **SCRS-S** | 7.5 | 12.6 | 8 | 16.9 |
| **SRS** | 4.5 | 27.5 | 8.8 | 17.5 |
