# Supplementary figures and images for: Unravelling the influence of mixed layer depth on chlorophyll-a dynamics in the Red Sea
Source: PLoS One. 2025 Mar 5;20(3):e0318214. doi: 10.1371/journal.pone.0318214 (PMC11882056; doi:10.1371/journal.pone.0318214)

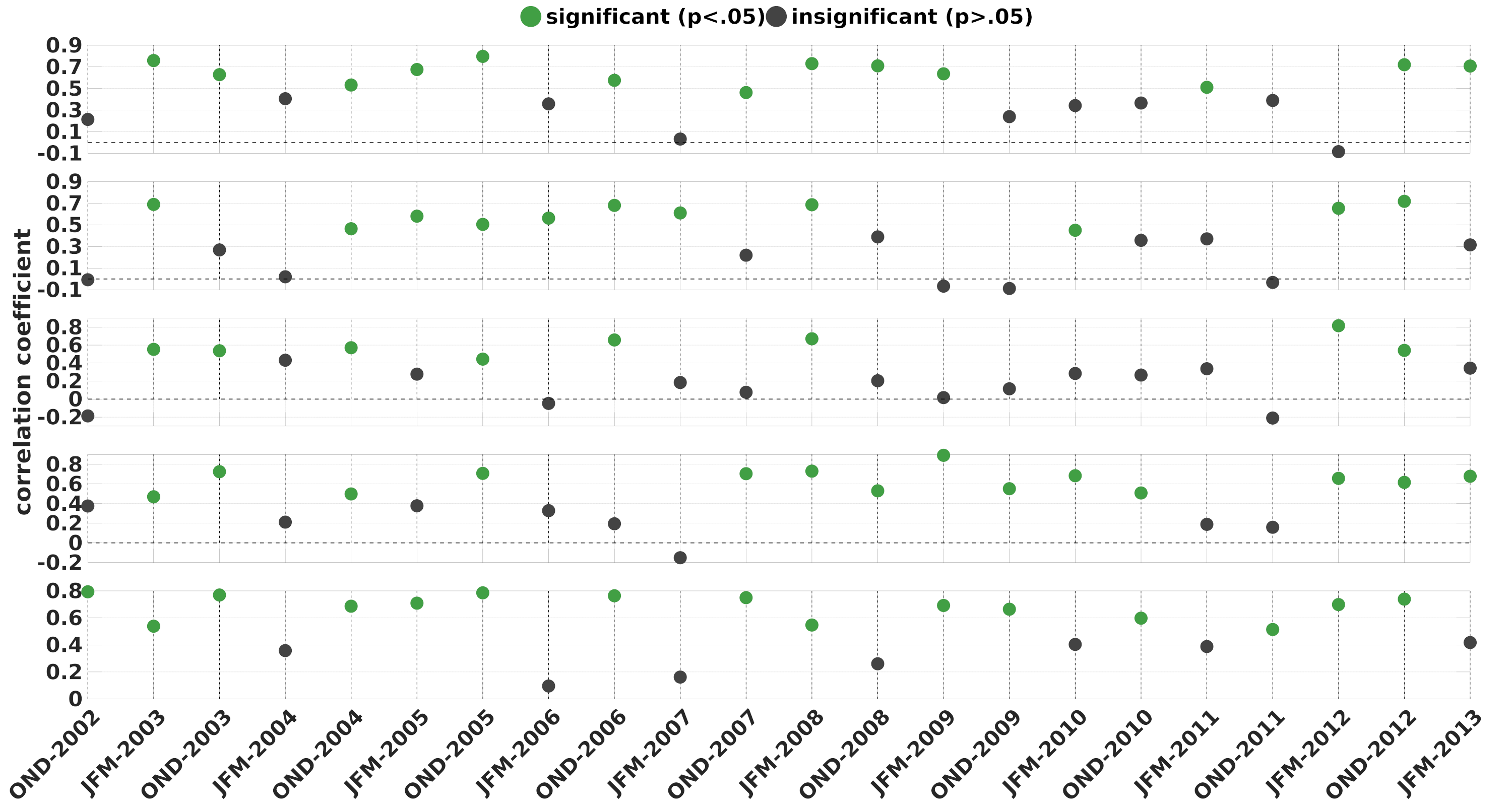

Supplement: S1 Fig — Green indices mark the significant correlation values (p < .05) while black indices the insignificant (p > .05). (TIF) [file pone.0318214.s003.tif]
